# Solution Structures of Two Different FRP-OCP Complexes as Revealed via SEC-SANS

**DOI:** 10.3390/ijms25052781

**Published:** 2024-02-28

**Authors:** Mina Hajizadeh, Maksym Golub, Marcus Moldenhauer, Olga Matsarskaia, Anne Martel, Lionel Porcar, Eugene Maksimov, Thomas Friedrich, Jörg Pieper

**Affiliations:** 1 Institute of Physics, University of Tartu, W. Ostwald Str. 1, 50411 Tartu, Estonia; mina.hajizadeh@ut.ee (M.H.); maksym.golub@ut.ee (M.G.); 2 Institute of Chemistry PC 14, Technische Universität Berlin, Straße des 17. Juni 135, 10623 Berlin, Germany; marcus.moldenhauer@tu-berlin.de (M.M.); friedrich@chem.tu-berlin.de (T.F.); 3 Institut Laue-Langevin, Avenue des Martyrs 71, CEDEX 9, 38042 Grenoble, France; matsarskaia@ill.fr (O.M.); martela@ill.fr (A.M.); porcar@ill.fr (L.P.); 4 Faculty of Biology, Lomonosov Moscow State University, 1–12 Leninskie Gory, 119991 Moscow, Russia; emaksimoff@yandex.ru

**Keywords:** photoprotection, orange carotenoid protein, fluorescence recovery protein, solution structure, small-angle neutron scattering, size exclusion chromatography

## Abstract

Photosynthetic organisms have established photoprotective mechanisms in order to dissipate excess light energy into heat, which is commonly known as non-photochemical quenching. Cyanobacteria utilize the orange carotenoid protein (OCP) as a high-light sensor and quencher to regulate the energy flow in the photosynthetic apparatus. Triggered by strong light, OCP undergoes conformational changes to form the active red state (OCP^R^). In many cyanobacteria, the back conversion of OCP to the dark-adapted state is assisted by the fluorescence recovery protein (FRP). However, the exact molecular events involving OCP and its interaction with FRP remain largely unraveled so far due to their metastability. Here, we use small-angle neutron scattering combined with size exclusion chromatography (SEC-SANS) to unravel the solution structures of FRP-OCP complexes using a compact mutant of OCP lacking the N-terminal extension (^∆NTE^OCP^O^) and wild-type FRP. The results are consistent with the simultaneous presence of stable 2:2 and 2:1 FRP-^∆NTE^OCP^O^ complexes in solution, where the former complex type is observed for the first time. For both complex types, we provide ab initio low-resolution shape reconstructions and compare them to homology models based on available crystal structures. It is likely that both complexes represent intermediate states of the back conversion of OCP to its dark-adapted state in the presence of FRP, which are of transient nature in the photocycle of wild-type OCP. This study demonstrates the large potential of SEC-SANS in revealing the solution structures of protein complexes in polydisperse solutions that would otherwise be averaged, leading to unspecific results.

## 1. Introduction

**Figure 1:**
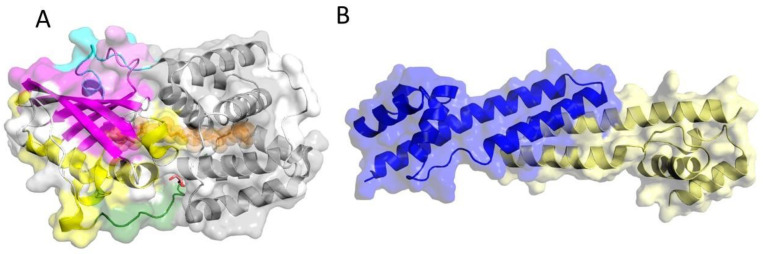
Crystal structures of OCP and the fluorescence recovery protein (FRP). (**A**) The structure of OCP^O^ (PDB:3MG1 [12]), NTD in gray, CTD in magenta and yellow, and hECN in orange. N-terminal extension (NTE) and C-terminal tail (CTT) are colored in cyan and purple, respectively. Flexible linker domain is highlighted in green. (**B**) Crystal structure of FRP homo dimer with two chains colored blue and yellow (PDB:4JDX [16]).

Light plays an ambivalent role for photosynthetic organisms [1]. On one hand, the absorption of solar radiation by photosynthetic pigment-protein complexes sustains photosynthesis, making it essential for survival [2], while on the other hand, high-light intensity may be destructive for the photosynthetic apparatus due to the accumulation of oxygen radicals [3,4,5]. Therefore, photosynthetic organisms have developed photoprotective mechanisms known as non-photochemical quenching (NPQ) to tackle this problem [6,7]. NPQ dissipates excess light energy in the form of heat to prevent damage to the photosynthetic apparatus [8]. In most cyanobacteria, excess light energy leads to the photoactivation of a soluble protein referred to as orange carotenoid protein (OCP) [9], which acts as a light intensity sensor and serves as a quencher of excess energy [10], thus controlling the energy flow from the light-harvesting phycobilisomes (PBSs) to the photosystems [11]. OCP-related genes can be found in the genome of many cyanobacteria, confirming its fundamental importance [11]. OCP consists of two main domains [12]: the N-terminal domain (NTD) with α-helical secondary structures and the C-terminal domain (CTD) with both α-helix and β-sheet structures [12]. In addition, there is an N-terminal extension (NTE) that attaches to the CTD in the compact, dark-adapted form of OCP, stabilizing this state. The structure of OCP is shown in Figure 1A. OCP has two roles: the sensory role is performed by the CTD, and the quenching role is carried out by the NTD [13]. These two domains enclose one keto-carotenoid, 3′-hydroxyechinenone (hECN) when purified from native cyanobacteria, which is bound noncovalently and is not accessible to the solvent [14]. OCP is also photoactive upon binding of the pigments echinenone (ECN) or canthaxanthin (CAN) when expressed in xanthophyll-producing *E. coli* strains. OCP also has a flexible linker domain (highlighted in green in Figure 1A) that connects the two NTD and CTD domains [13]. The NTE also carries an α-helical secondary structure element (αA helix), which plays an important role in the conformational change during photoactivation [15].

In the case of excess light energy, the orange dark-adapted form OCP^O^ undergoes a transformation to a red active state (OCP^R^) with a low quantum yield (0.2%) [17,18,19]. The photocycle of OCP has different intermediate steps [20,21,22], encompassing the breaking of the hydrogen bonds between the protein and the keto-carotenoid, a relocation of the carotenoid into the NTD, the detachment and unfolding of the NTE, and, finally, the separation of the two domains to reach the extended conformation of OCP^R^. In 2015, Leverenz et al. [23] reported the structure of the NTD binding canthaxanthin (CAN) (PDB:4XB4). Notably, the carotenoid’s position in the NTD structure significantly differs from its location in OCP^O^. The NTD with CAN exhibits all the molecular characteristics of OCP^R^, including a red-shifted absorption spectrum, the ability to bind to PBS, and fluorescence quenching activity. Moreover, experiments involving mass spectrometry [24] and X-ray scattering in solution [25] enabled the observation of the structural rearrangement of OCP^R^ into two separated domains. Collectively, these findings suggest that the disruption of the two hydrogen bonds within the CTD, specifically those involving the 4-ketolated β1-ring and Tyr201 and Trp288, leads to the carotenoid’s migration into the NTD, resulting in domain separation between the NTD and CTD [26,27]. Recent studies have also reported additional steps that occur before domain separation, including the detachment of the NTE and the C-terminal tail (CTT) from the CTD [24,25,28]. Once released, the C-terminal tail may change its position and block the carotenoid binding site of the conformation, which remains empty in OCP^R^.

Despite these advances, the high-resolution structure of OCP^R^ remained elusive until recently. Then, cryo-electron microscopy provided structural insights into the OCP^R^ structure within the quenched OCP-PBS complex from *Synechocystis* sp. PCC 6803 at a resolution of 2.7 Å [29]. The observed OCP^R^ structure generally aligns with those in previous studies, featuring distinct domains and placing the carotenoid within the NTD. Additionally, it highlights that the quenching of the PBS antenna complex necessitates the binding of a dimer of OCP molecules in the OCP^R^ state [29]. 

The low-resolution solution structures of OCP^O^ and OCP^R^ had been investigated earlier using small angle x-ray and neutron scattering (SAXS and SANS) [20,25]. SAXS revealed significant alterations in the overall tertiary structure, with complete domain separation observed during photoactivation [25]. Notably, in SANS experiments, OCPs, whether in their active or dark-adapted states, exhibited dimerization even at low concentrations [20].

The picosecond molecular dynamics of OCP were investigated via quasielastic neutron scattering (QENS) [30,31]. The results revealed that upon the illumination and separation of the two OCP protein domains [30], exterior residues gain more motional degrees of freedom [30] and bind more water molecules due to the effectively larger surface. This additional motional freedom may provide channels for non-radiative decay and thus directly support NPQ. 

The back conversion of OCP^R^ to the dark-adapted ground state may occur spontaneously. However, the interaction of the fluorescence recovery protein (FRP) with OCP was shown to accelerate the back conversion. Despite previous efforts, the mechanisms of these molecular events and OCP^R^-FRP complex(es) are still obscure, while the main barrier is the metastability of OCP^R^ and its complexes with PBS and FRP [32]. FRP facilitates the separation of OCP^R^ from PBS and acts as a scaffold to restore the dark-adapted form OCP^O^ [28]. FRP is a dimeric protein with an α-helical secondary structure [16]. As seen in Figure 1B, it has a high binding affinity to OCP^R^ with a K_d_ of around 3 µM [32], and it is tightly bound to OCP^R^, but its affinity to OCP^O^ is lower with a K_d_ of around 35 µm [32]. It has been shown that the FRP binding site is located on the OCP’s CTD [16]. Consequently, FRP can interact with OCP^O^, especially in constructs lacking the NTE, and with an isolated CTD [32]. The interaction between FRP and OCP^O^ lacking an NTE has been studied before, and the 2 FRP:1^∆NTE^OCP^O^ complex structure has been reported based on SAXS [32] and SANS [33] experiments. In addition, evidence for other stoichiometries of FRP^∆NTE^OCP^O^ complexes have been presented, but no structures for these complexes have been determined so far [32]. Moreover, time-resolved spectroscopy has been used to shed light on the dynamics of the interaction between FRP and OCP [34]. 

Size exclusion chromatography–small-angle neutron scattering (SEC-SANS) is a powerful method for studying solution structures and protein–protein interactions [35,36]. This technique combines the strengths of two established analytical methods: SEC and SANS. Here, SEC allows the separation of proteins of different molecular masses present in a potentially polydisperse sample solution prior to SANS. The sample eluting from the SEC column is immediately illuminated through a neutron beam. Without SEC, the SANS signals of various constituents of the polydisperse solutions would be averaged and difficult to deconvolute. In contrast, using SEC-SANS fractions of different molecular masses can be analyzed separately, revealing their sizes and shapes in solution [37,38]. SANS has been widely used in various protein systems, including studies on protein folding [39], ligand–protein interactions [40], and the structural analysis of protein complexes, providing valuable insights into the mechanisms of biological processes. One advantage of SANS is its immunity to radiation damage, a frequent issue encountered in synchrotron SAXS experiments [41,42]. In photosynthesis research, SAXS and SANS techniques are used to determine the solution structures of the major antenna complex LHC II of green plants [43,44], the bacterial antenna complex LH2 [45], photosystem I (PSI) [46,47,48], and photosystem II (PSII) [49]. Therefore, SANS appears to be the technique of choice for studying the interactions of FRP and OCP complexes under nearly physiological conditions in solution [48]. 

In the present study, we employed SEC-SANS to investigate the FRP-OCP complex formation, because OCP was shown to be prone to form various coexisting oligomers [20,32,50]. In addition, it can be expected that different stoichiometries of FRP-OCP complexes may exist in solution [32]. We use a specific orange variant of OCP lacking the NTE and featuring a compact structure similar to the dark-adapted state of OCP, which is referred to as ^∆NTE^OCP^O^ and was shown to bind wild-type FRP [33]. We did not attempt selective deuteration as demonstrated before by Golub et al. [33], because rather subtle structural changes in the individual proteins were observed within the whole FRP-OCP complex. SEC-SANS allowed us to identify different types of OCP-FRP complexes that would otherwise be averaged in a conventional SANS experiment. We anticipate that the observed ^∆NTE^OCP^O^-FRP complexes represent potential transient intermediates during the back conversion of OCP to its dark-adapted state, the presence of which is exacerbated by the absence of the NTE.

## 2. Results

**Figure 2:**
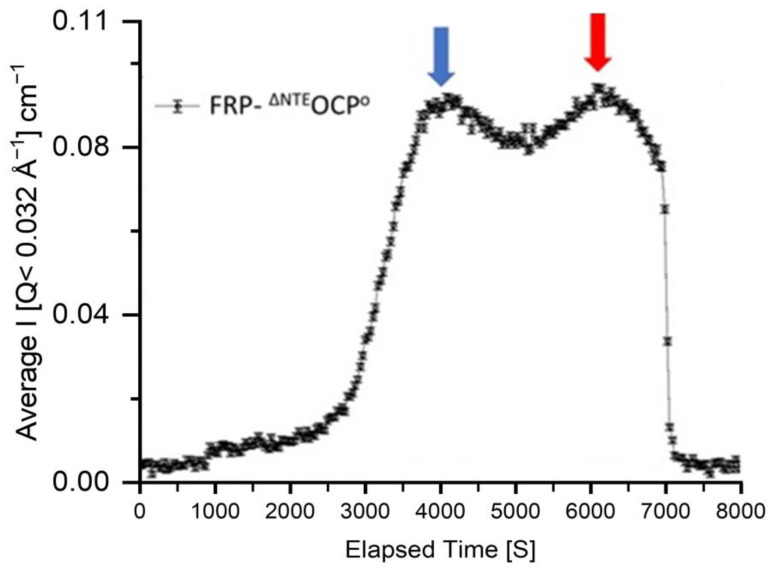
Scattergram obtained in a SEC-SANS measurement at D22 indicating the presence of two different fractions of the FRP-^∆NTE^OCP^O^ complex. The total protein concentration before injection was about 5 mg/mL. Different colors are necessary to show the high MW (molecular weight ) in blue and low MW in red. The SANS data obtained for each of the fractions are shown in Figure 3.

**Figure 3:**
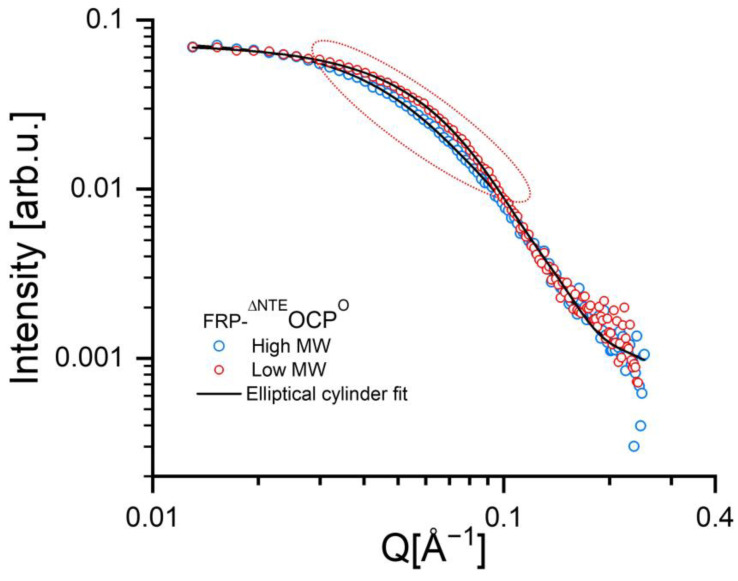
SANS data of FRP-^∆NTE^OCP^O^ complexes. The blue circles represent the SANS data of the (elongated) high MW and the red circles those of the low MW (compact) in the SEC experiment, respectively, measured at room temperature using D22. The color code is the same as that used for the arrows in Figure 2. Black solid lines correspond to the fitting curves of using an elliptical cylinder model (see Table 1 for fitting parameters).

SEC-SANS Experiment: The SEC-SANS measurements of FRP-^∆NTE^OCP^O^ complexes were carried out at D22 at room temperature (see Figure 2 and Figure 3). SEC is one of the mildest protein separation methods that can separate proteins based on their size. Once interfaced with SANS as available on D22, SEC-SANS permits the separate analysis of the SANS data of protein populations, which would otherwise be hidden in polydisperse solutions [51]. Such a case is indicated in the data shown in Figure 2, where two peaks are observed in the SEC data analysis (Figure 2). Another benefit is that in SEC-SANS experiments utilizing the same flow cell for both the buffer and sample, background subtraction is notably more precise. Inaccurate background subtraction from SANS data could potentially lead to erroneous calculations of size parameters [52].

The peak positions are highlighted by blue and red arrows (Figure 2) corresponding to the higher and lower molecular weight components, respectively. Below, the latter components will be referred to as the high MW (molecular weight)in blue and low MW in red. 

The SANS data of the high MW and low MW are shown in Figure 3 and reveal noticeable differences in the intermediate Q-range between 0.04 and 0.09 Å^−1^. Here, the SANS curve of the high MW sample is slightly shifted toward lower Q-values, indicating more extended objects than in the low MW. Due to the use of SEC-SANS, the data also do not indicate any undesired effects due to sample aggregation (see e.g., [44]). In summary, the FRP-^∆NTE^OCP^O^ solution comprises two distinct populations that can be separated effectively by applying SEC-SANS.

**Figure 4:**
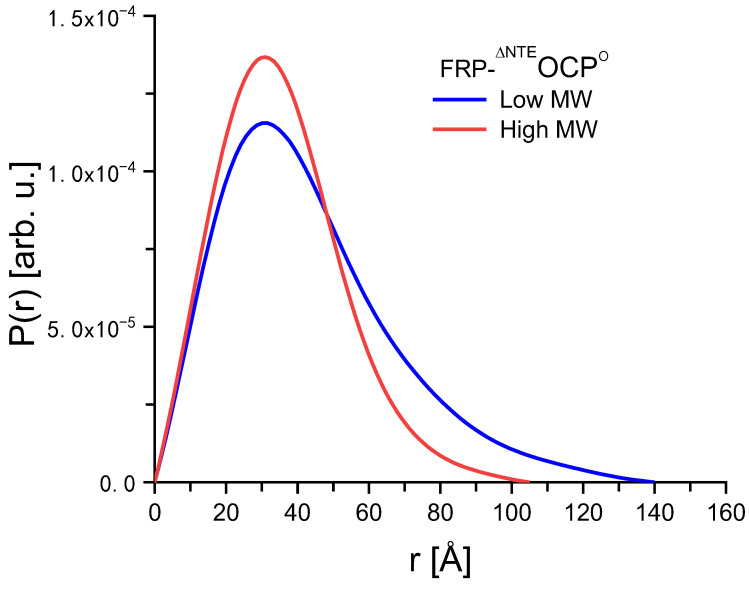
Comparison of P(r) functions calculated from the SANS data shown in Figure 3 for the high MW (blue line) and the low MW (red line) of a FRP-^∆NTE^OCP^O^ complex sample.

**Figure 5:**
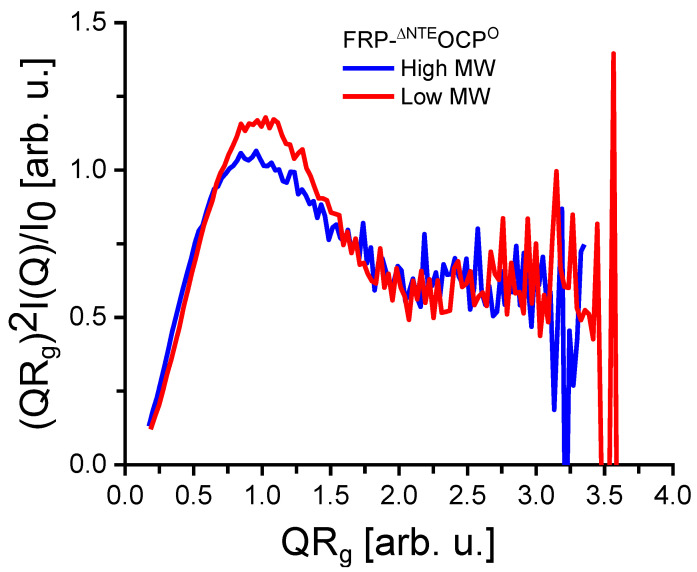
Kratky plot calculated from the SANS data shown in Figure 3 for the high MW (blue line) and the low MW (red line) of a FRP-^∆NTE^OCP^O^ complex sample.

Based on the SANS data shown in Figure 3, the solution structures of the FRP-^∆NTE^OCP^O^ complexes can be characterized by the P(r) function given in Figure 4. The D_max_-values obtained are about 140 and 102 Å for the high MW and low MW, respectively. An analysis of these results using models of cylinders indicated that the high MW is an elongated molecule with larger radius than the low MW, which appears to be more globular. It is also essential to note that according to the Kratky plots shown in Figure 5, there is no sign of unfolding. Unfolded (highly flexible) proteins are characterized by a plateau in the Kratky plot at higher Q-values, while compact proteins are expected to exhibit a bell-shaped, nearly Gaussian peak, as visible in Figure 5. At the same time, the P(r)function tends toward a slightly smaller D_max_ value for the low MW Figure 4. This is again indicative of two protein fractions with different dimensions.

The presence of two different types of FRP-^∆NTE^OCP^O^ complexes is also reflected in the results of a model-dependent fit using the shape of an elliptical cylinder. The fits are shown as full black lines in Figure 3, and the fit parameters are summarized in Table 1. The dimensions of the low-MW, the more compact fraction, correlate very well with the values from previous experiments with P(r) function of 130 Å [32] and 110.4 Å [33], corresponding to a 2 FRP-1 ^∆NTE^OCP^O^ stoichiometry. The P(r) function value is slightly lower than 130 Å, from [32] (representing a disulfide-trapped FRP dimer), which could be due to the extra mutation FRP. The elliptical cylinder’s length for the low MW is equal to 73 ± 4 Å with a minor elliptical radius equal to 14.6 ± 4.0 Å, so that the combined lateral size of the of the complex coincides well with the dimensions of a FRP dimer binding a monomeric ^∆NTE^OCP^O^ [32]. In contrast, the high MW exhibits a considerably larger length of 140 Å with an ellipsoidal radius of 13.5 ± 6.0 Å suggesting again a structural difference between the two fractions of FRP-^∆NTE^OCP^O^ complexes. We note that the values for the ellipsoidal radii are similar within the experimental error for both fractions, while the differences in cylinder length are striking. The parameters for the high-MW, the elongated fraction appear to be consistent with an ^∆NTE^OCP^O^-2FRP-^∆NTE^OCP^O^complex, i.e., with a 2:2 stoichiometry, see also below.

In this regard, it is essential to note that the ^∆NTE^OCP^O^-2FRP-^∆NTE^OCP^O^ (2:2) complex has been suggested to be a crucial intermediate in the OCP photocycle [32]. This complex may be a pivotal bridge between the initial interaction of OCP with dimeric FRP and the subsequent formation of a 1:1 complex. Suchan intermediate, would play a central role in the efficient deactivation of OCP. 

**Figure 6:**
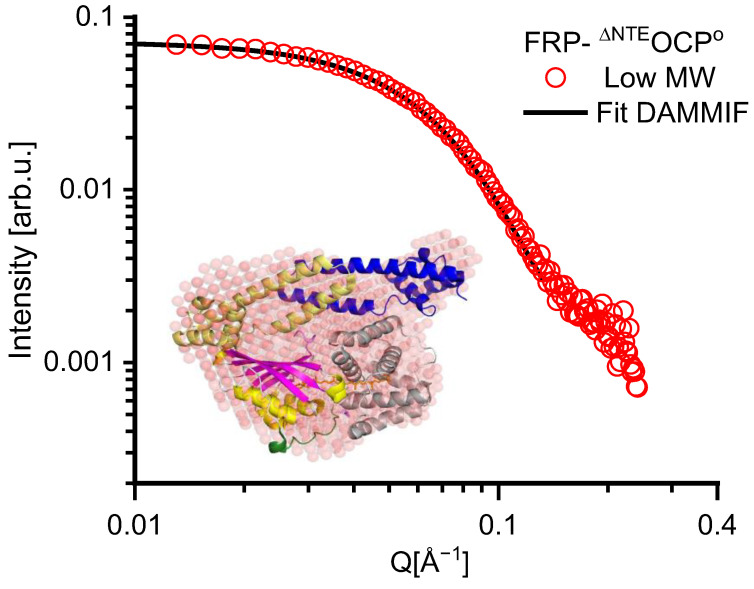
Low-resolution structure (red spheres) obtained from the SANS data (red circles) of the low MW, compact fraction of FRP-^∆NTE^OCP^O^ complexes at room temperature. The fit (black line) corresponds to the reconstructed solution structure of OCP depicted by red spheres in the inset and was obtained using the software package DAMMIF (https://www.embl-hamburg.de/biosaxs/manuals/dammif.html, accessed on 22 December 2023). The fitting was restricted to the Q range of 0 ≤ Q Å^−1^ ≤ 0.13. The crystal structure of monomeric OCP^O^ (PDB:3MG1) with removed NTE and dimeric FRP (PDB:4JDX) is shown in the inset for comparison and has the same color code as Figure 1.

Low-resolution Solution Structure of the low MW. The solution structures of the FRP-^∆NTE^OCP^O^ complex samples were generally obtained from the SEC-SANS data, which also largely diminish the impact of protein aggregation. The SANS data of the low-MW FRP-^∆NTE^OCP^O^ sample are shown as red circles in Figure 6. The solution structure resulting from the DAMMIF reconstruction of the sample is depicted by red spheres in the inset of Figure 6, (see Table 1 for a complete set of parameters). The DAMMIF fitting curve is shown as a black line in Figure 6. In assuming the presence of a 2:1 stoichiometry, the corresponding crystal structure composed of monomeric OCP^O^ (PDB:3MG1 after the NTE removal) and dimeric FRP (PDB:4JDX) is shown in the inset of Figure 6 for comparison. A comparison reveals that the structure reconstituted from the SANS data widely resembles the size and shape of the FRP-^∆NTE^OCP^O^ as a 2:1 complex. It has to be mentioned that previous studies reported the presence of 1:1 and 2:2 complexes, and a solution structure of the 2:1 complex seems to be the major species in solution [32]. 

**Figure 7:**
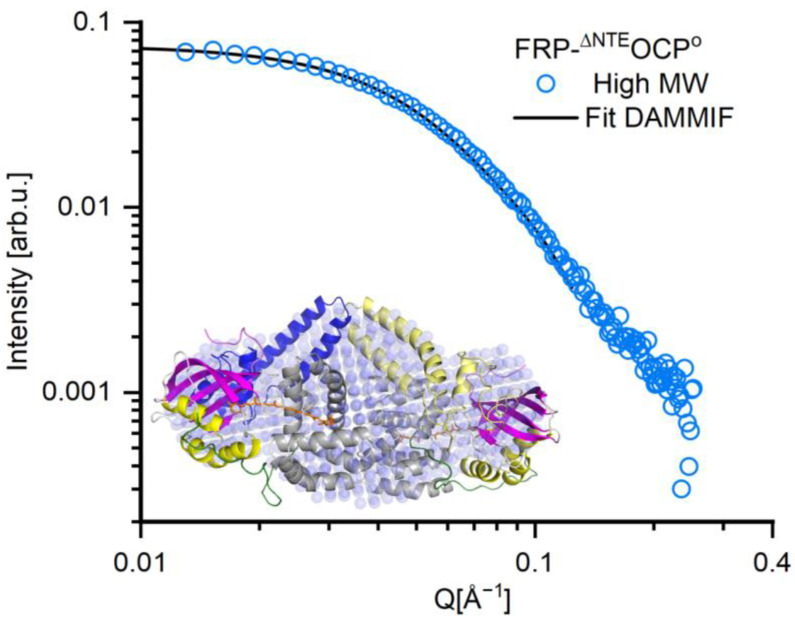
Low-resolution structure (blue spheres) obtained from the SANS data (blue circles) of the high-MW, elongated fraction of FRP-^∆NTE^OCP^O^ complexes at room temperature. The fit (black line) corresponds to the reconstructed solution structure of OCP depicted by blue spheres in the inset and was obtained using the software package DAMMIF. The fitting was restricted to the Q range of 0 ≤ Q Å^−1^ ≥ 0.13. The crystal structure of two OCP^O^ (PDB:3MG1) with removed NTE and dimeric FRP (PDB:4JDX) is shown in the inset for comparison and has the same color code as Figure 1.

Low-resolution Solution Structure of the high MW. The SANS data of the high MW is shown in Figure 7 by blue circles. The fit function determined for the high MW is also shown as a black line in Figure 7. The fit obtained using DAMMIF (see Table 1 for a complete set of parameters) shows an excellent agreement with the data in Figure 7 and corresponds to the reconstituted solution structure of the high MW sample given by blue spheres in the inset of Figure 7. In contrast to the low MW (see Figure 6), the reconstituted structure of the high MW of FRP-^∆NTE^OCP^O^ appears elongated. Here, we assume that this elongation may be due to the presence of a second ^∆NTE^OCP^O^ resulting in an overall 2FRP:2^∆NTE^OCP^O^ stoichiometry. This is consistent with the results of the elliptical cylinder model (see Table 1) and with those of previous studies [32].

**Figure 8:**
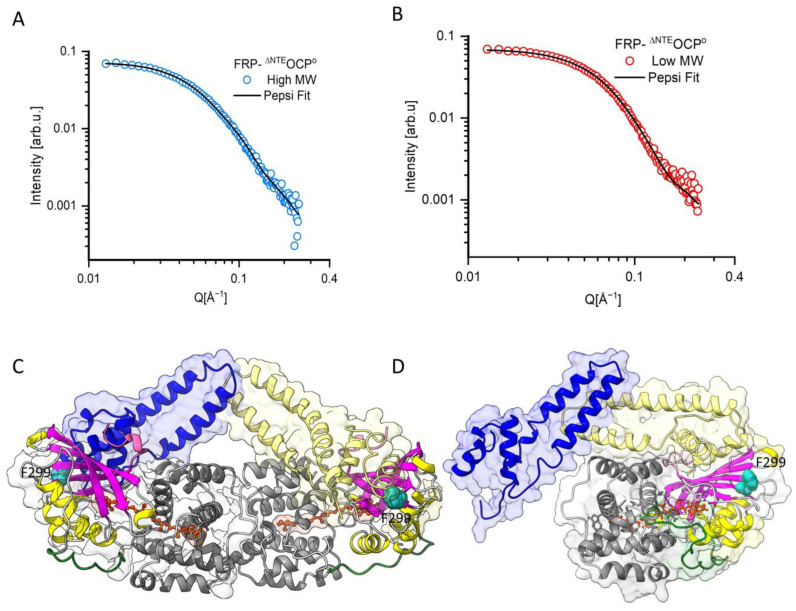
Atomistic PepsiFlex modeling based on the SANS data of the high-MW (blue circles in panel (**A**)) and low-MW (red circles in panel (**B**)) fractions of FRP-^∆NTE^OCP^O^ complexes at room temperature. Panel (**C**) shows the 2:2 FRP-^∆NTE^OCP^O^ complex and Panel (**D**), the 2:1 FRP-^∆NTE^OCP^O^ complex obtained from the SANS data in panels (**A**,**B**), respectively, using PepsiFlex based on the crystal structures of OCP^O^ (PDB:3MG1) with removed NTE and of FRP (PDB:4JDX) and have the same color code as Figure 1. The residue Phe299, highlighted in red, is known to be part of the binding site for FRP.

Atomistic model of FRP-^∆NTE^OCP^O^: More detailed atomistic structural models are shown in panels C and D of Figure 8 as obtained using the program Pepsi-SANS in its ‘‘FlexFit’’ option. Pepsi offers a significant advantage over DAMMIF since it integrates high-resolution information from crystallographic structures. This advantage can be crucial in many scientific and research contexts, e.g., in carrying out complementary molecular dynamic simulations for the structural intermediates of a protein that have not been crystallized. Pepsi-SANS can quickly generate models along the slowest normal modes computed for the starting structure and their scattering curves and relate them with experimental data (after smearing them through the instrumental resolution).

Although PepsiFlex fits can be based on existing high-resolution structures, the question of the exact formation of the FRP-OCP complexes has to be addressed before. A previous study [53] indicated that the ability of FRP to facilitate the back conversion of OCP to its dark-adapted state is compromised when a mutation is introduced at position 299 (Phe299) of the CTD of OCP (shown in red in Figure 8D). Therefore, this residue might be essential for the proper binding of FRP to facilitate the OCP back conversion. Consequently, the CTD was used as the connection point between FRP and OCP in the Pepsi modeling presented here [32,53]. As reported in our previous paper [33], we considered two distinct rigid blocks within the FRP-^∆NTE^OCP^O^ complex. The first block includes the ^ΔNTE^OCP^O^ protein, consisting of rigid amino acid residues 30–300, and the second block is in both FRP monomers, amino acid residues 14–100, which are in contact with OCP. Experimental SANS profiles for both FRP-^∆NTE^OCP^O^ fractions are plotted with the best fits of the atomistic models by PepsiFlex as black lines in Figure 8A,B. The quualityof the fit to the experimental data was evaluated by calculating the reduced χ^2^ (χ^2^= 0.86, 0.66 for high MW and low MW, respectively). The Pepsi modeling curves shown in Figure 8A, B exhibit excellent agreement with the experimental data. Employing the PepsiFlex fit method, we observed the same reorientation of the FRP monomers relative to each other (FRP monomers exhibited a tilt of approximately 30 degrees toward the second FRP monomer, accompanied by a slight increase in the bend angle from 130 to 141 degrees; see Figure 8), as in our previous paper [33].

In summary, the excellent agreement of the PepsiFlex fits with the experimental data visible in Figure 8 supports the assignment of the two protein fractions visible in the present SEC-SANS data to 2:1 and 2:2 stoichiometries of FRP-^∆NTE^OCP^O^ complexes. We anticipate that our solution structure of the 2:2 complex between the FRP-^∆NTE^OCP^O^s may represent an intermediate in the OCP photocycle in the presence of FRP, which appears here because of the use of a variant of OCP lacking the NTE, but it may be of transient nature in the case of wild-type OCP.

## 3. Discussion

In previous work [28,32], as well as in the present study, it has been shown that ^∆NTE^OCP^O^ can bind FRP as a 2FRP:1^∆NTE^OCP^O^ complex, which is consistent with the importance of the unfolding of the NTE during the OCP photocycle in order to unblock the binding site for FRP attachment. Sluchanko et al. [32] reported a structure of the 2:1 complex based on SAXS data that is consistent with our model shown in Figure 8B,D. Our SEC-SANS data are consistent with the occurrence of a ^∆NTE^OCP^O^-2FRP-^∆NTE^OCP^O^ complex and enabled us to propose a potential solution structure for this 2:2 complex for the first time (Figure 8A,C). The latter structure was not observed in the previous study by Golub et al. [33].

Tsoraev et al. [34] and Andreeva et al. [50] discussed the importance of the oligomeric (monomeric/dimeric) state of OCP^R^ for the adaptation of the OCP photocycle to different environmental conditions. They suggested that the dimeric OCP^R^ can play a functional role, owing to its sustained presence resulting from a slow relaxation rate. This is also supported by recent high-resolution structural data presented by Dominguez-Martin et al. [29], which reveals interactions between the dimeric OCP^R^ form and the core of the PBS. Golub et al. [33] demonstrated that two FRP dimers interact simultaneously with the functional dimeric OCP^R^ complex. However, it is essential to note that these interactions induce conformational changes within the FRP structure [33]. Sluchanko et al. [32] discussed the presence of 2:2 and 2:1 complexes with potential roles as intermediates of the OCP back conversion to its dark-adapted state in the presence of FRP. It was observed that monomeric FRP displayed a limited binding affinity to the photoactivated OCP. Instead, OCP appeared to recruit dimeric FRP preferentially. Upon interaction with OCP, dimeric FRP is assumed to undergo a subsequent monomerization process, resulting in the formation of a 1:1 complex. Notably, this transition from dimeric to monomeric FRP in the presence of OCP seemed to be facilitated by the transient formation of an ^∆NTE^OCP^O^-2FRP-^∆NTE^OCP^O^ complex. This complex appears to be characterized by the interaction between the two FRP head domains and may play a pivotal role in significantly enhancing the efficiency of FRP, particularly at elevated OCP levels. Our data show that the mutant ^∆NTE^OCP^O^ lacking the NTE permits the observation of such otherwise transient intermediates as stable complexes of FRP with a compact OCP form. Eventually, OCP^R^ is converted back to the inactive OCP^O^ state facilitated by FRP. Within this back conversion, a 2:2 complex may be formed directly from dimeric OCP^R^, or another free OCP^R^ may bind to the FRP-OCP^R^ complex and assemble. This 2:2 complex with OCP^R^s transforms into a 2:2 complex with OCP^O^s, followed by the dissociation of the first OCP^O^ and formation of a 2:1 complex through steric clashes. The exact function of the 2:2 complex between FRP and OCP^O^ remains elusive; however, it is evident that this intermediate plays a critical role in photoprotection. A scheme of the OCP photocycle in the presence of FRP highlighting the processes discussed above is available from Tsoraev et al. [34].

## 4. Materials and Methods

### 4.1. Sample Preparation

The sample preparation was described in detail by Golub et al. (2023) [33]. Briefly, the genes of OCP (*slr1963*) and FRP (*slr1964*) from *Synechocystis* sp. PCC 6803 were optimized for expression in *E. coli* and cloned into the pRSFDuet-1 vector (Merck Millipore, Burlington, Massachusetts, USA). The ^ΔNTE^OCP^O^ variant was obtained by introducing a human rhinovirus 3C protease cleavage site (LEVLFQ/GP) at Pro13 in OCP, so that the resulting ^ΔNTE^OCP^O^ sequence after the cleavage of the His-tag starts with GP-13-NTLAA and is lacking the NTE. FRP and ^ΔNTE^OCP^O^ were expressed in *E. coli*. Proteins were isolated, purified, and finally concentrated to 500 µL, loaded on a Superdex™ 200 Increase 10/300 (Cytiva, Marlborough, MA, USA), and eluted using phosphate-buffered saline. The proteins were stored at −80 °C.

### 4.2. SANS Experiment

SANS measurements were conducted with a D22 SANS instrument at Institut Laue Langevin, Grenoble, France, at a Q range from 0.03 to 0.25 Å^−1^ at a neutron wavelength of 6 Å ± 10% and using a two-dimensional (96 cm × 96 cm) detector at a sample-to-detector distance of 8 m. A quartz-based sample cell was integrated into the in situ size exclusion chromatography (SEC) system equipped with a Superdex 200 Increase 10/300 GL SEC column (Cytiva, Marlborough, MA, USA) [54]. The experimental temperature was maintained at 25 °C, and the flow rate was set at 0.5 mL/min prior to the appearance of the initial elution peak, decreased to 0.07 mL/min thereafter. Typically, sample injections were 250 µL in volume, with a protein concentration of 5 mg/mL. 

Using an exposure time of 30 s, SANS data were continuously recorded during the SEC elution. All samples (including water and buffers) were measured in quartz cuvettes with 1 mm optical path length. Data were reduced according to standard procedures using the GRASP (Graphical Reduction and Analysis SANS Program) [55], including thickness and transmission normalization as well as solvent background subtraction. 

### 4.3. Data Analysis

The differential scattering cross-section per unit volume, [dσ/dΩ], was obtained from the SANS experiment, which is the number of neutrons scattered into a unit solid angle per path length. The absolute scattering intensity for a dilute monodisperse solution is given as follows [48]:

(1) $$I\left(Q\right)=\frac{d\sigma }{d\Omega }=n\Delta \rho^{2}V^{2}P\left(Q\right)S\left(Q\right),$$

where Q is the scattering vector $Q=\frac{4\pi }{\lambda_{0}}\sin\theta \;$ with λ_0_ being the neutron wavelength, and θ is the scattering angle. Furthermore, the protein molecule’s particle number density in solution is denoted by n, and V is the protein volume. The difference in the scattering length density of the protein molecules and the solvent is represented by $\Delta \rho^{2}$. P(Q) is the form factor, which is a function of the averaged shape and size of the scattering particles. S(Q) is the structure factor equal to the unity for diluted solutions without particle interaction. The data were analyzed by comparing the scatterings from different models to the experimental data using SasView [56].

The pair distribution function P(r), with r being the distance between the paired scattering elements in the molecule under study, is related to the scattering intensity given by Equation (2) [57]:

(2) $$I\;\left(Q\right)=4\pi \int_{0}^{D_{max}}P\left(r\right)\frac{\sin\left(Qr\right)}{Qr}\;dr,$$

which can be derived from the Debye formula [58]. P(r)values can be calculated from the entire scattering curve I(Q), in the range of Q from zero to Q_max_, through an indirect Fourier transform (IFT), where D_max_ is the maximum dimension of the protein molecule:

(3) $$P\left(r\right)=\frac{r^{2}}{2\pi^{2}}\;\int_{0}^{\infty }\frac{Q^{2}I(Q)\sin Qr}{Qr}dQ.$$

An analysis was carried out using the GNOM routine [59]. The fitting also provided the radius of gyration (R_g_), the second moment of P(r), and the forward scattering, I(0). R_g_ can be described as follows:

(4) $$R_{g}^{2}=\frac{\int_{0}^{D_{max}}r^{2}P\left(r\right)dr}{2\int_{0}^{D_{max}}P(r)dr}.$$

The model utilized in this study is the elliptical cylinder, which was averaged over all possible orientations and is defined according to [60], which is as follows:

(5) $$P_{cylinder}\left(Q\right)=\frac{S_{cylinder}}{V_{cyl}}\int_{0}^{1}\Psi_{ec}(Q,a\sqrt{1-x^{2}})j_{0}^{2}(\frac{QLx}{2})dx,$$

where S_cylinder_ is a scaling factor; V_cyl_, the particle volume; α, the minor radius of the elliptical cross-section; and L is the length of the elliptical cylinder. The zeroth-order Bessel function is equal to $j_{0\frac{\sin\left(t\right)}{t}}$. Additionally, the function Ψ_ec_ (Q, a) is given as follows:

(6) $$\psi_{ec}\left(Q,a\right)=\frac{1}{\pi }\int_{0}^{\pi }\Lambda_{1\;}^{2}\left[Qa{\left(\frac{1+v^{2}}{2}+\frac{1-v^{2}}{2}\cos\left(y\right)\right)}^{\frac{1}{2}}\right]dy.$$

Here, ν is the ratio between the major and minor radii of the elliptical cross-section. The function Λ_1_ is defined as $\;\frac{2j_{1}(t)}{t}$*,* where *j*_1_ is the first-order Bessel function $j_{1}=\frac{\sin\left(t\right)-t\cos\left(t\right)}{t^{2}}$.

The reconstructed low-resolution three-dimensional structural models were obtained from the P(r) function using the DAMMIF program [61] based on a Monte Carlo approach without any a priori information about the molecule’s structure. About 20 raw three-dimensional protein models were generated and averaged for each sample fraction to obtain a final single three-dimensional protein model. The final models were obtained by imposing P2 and P1 symmetry restrictions for the high MW and low MW, respectively. The scattering intensity for the dummy model was calculated as follows [61]:

(7) $$I\left(Q\right)=2\pi^{2}\sum_{i=0}^{\infty }\sum_{m=-l}^{l}{\left|A_{lm}(Q)\right|}^{2},$$

where the amplitude A_lm_ is calculated as follows [61]:

(8) AlmQ=il2π2  va ∑j=1Xj=1Mjl Qrj Ylm*(ωj).

Here, (r_j_, ω_j_) are polar coordinates, and $Y_{lm}$ is the spherical harmonics.

To go beyond the approach using DAMMIF, following our previous study [33], we prepared an atomistic molecular model and calculated the SANS curve using Pepsi-SANS to obtain a more refined picture. The method uses a multipole-based scheme initially proposed by Stuhrmann [62]. It is swift, allows for explicit and implicit hydrogens, and permits the specification of the sample’s deuteration level, the buffer, and the exchange rate of labile hydrogens [63]. Solution structure modeling was carried out using the software package Pepsi (Polynomial Expansions of Protein Structures and Interactions) (https://pepsi.app.ill.fr/, accessed on 22 December 2023) based on the crystal structures of OCP^O^ (PDB: 3MG1 and 4XB5 after the removal of the NTE) and of FRP (PDB: 4JDQ, chains B and D). The Pepsi program applies the Nyquist–Shannon–Kotelnikov sampling theorem, ensuring that the multipole expansion order was adjusted according to the model’s size and the resolution of the experimental data. To enhance the execution speed of the Pepsi method, cubic spline interpolation was implemented. 

In the FlexFit mode, the Pepsi program employs SAXS/SANS-based body modeling of protein complexes. In this mode, only a specific portion or domain of a protein can be defined as rigid. It can be repositioned relative to the initial position for the optimal fitting of the small-angle scattering data. This means that it does not substitute flexible protein regions with interconnected chains of dummy residues attached to rigid domains. During the final stage of analysis, the Cryson program [64] was used to verify the solution structure models of the OCP-FRP complexes generated by the Pepsi program.

## 5. Conclusions

We used SEC-SANS to investigate the solution structures of FRP-OCP complexes using a compact variant of OCP lacking the N-terminal extension (^∆NTE^OCP^O^) and wild-type FRP. The results indicate the simultaneous presence of two different fractions, one being more compact and one with elongated structure. Since the occurrence of 2:2 and 2:1 FRP : ^∆NTE^OCP^O^ complexes in solution was known, it appeared reasonable to associate the two observed fractions with the latter complex stochiometries. On this basis, ab initio low-resolution structures and homology models derived from available crystal structures can be provided for both types of complexes. It is assumed that these structures represent the intermediate states of the back conversion of OCP from the photoactivated to its dark-adapted state in the presence of FRP, which are of transient nature in the photocycle of wild-type OCP.

## Figures and Tables

**Table 1:** Parameters obtained by fitting the FRP-^∆NTE^OCP^O^ complex SANS data shown in Figure 3 using an elliptical cylinder model.

	FRP-^∆NTEO^CP^O^ High MW	FRP-^∆NTEO^CP^O^ Low MW
Guinier Analysis		
I(0)	0.075 ± 0.00044	0.072 ± 0.00043
R_g_(Å)	32.08 ± 0.033	27.04 ± 0.027
QR_g_ Range	0.56 < QR_g_ < 1.29	0.35 < QR_g_ < 1.26
P(r) Analysis		
I(0)	0.075	0.072
R_g_(Å)	34.04 ± 0.08	27.51 ± 0.07
D_MAX_(Å)	140	102
Ranges of data points taken for the fit	3–113	1–115
Kratky plot	folded	folded
Parameters of elliptical cylinder model		
Minor radius (Å)	13.49 ± 6.29	14.64 ± 0.35
Length (Å)	100 ± 10.44	73 ± 1.32
Axis ratio	2.06 ± 1.48	2.07 ± 0.98
Scaling factor	0.001 ± 0.0003	0.001 ± 0.00001
X^2^	0.74	0.78

## Data Availability

The data presented in this study are available on request from the corresponding author on reasonable request.
